# Aerobic exercise improved liver steatosis by modulating miR-34a-mediated PPARα/SIRT1-AMPK signaling pathway

**DOI:** 10.1371/journal.pone.0333872

**Published:** 2025-11-12

**Authors:** Baoai Wu, Zhibin Zhang, Chong Xu, Jinfeng Zhao

**Affiliations:** School of Physical Education, Shanxi University, Taiyuan, China; Université Clermont Auvergne - Faculté de Biologie, FRANCE

## Abstract

MicroRNA-34a (miR-34a) was closely associated with liver steatosis. However, the link between changes in miR-34a and the progression of liver steatosis remained unclear. In the work, sixty mice were randomly and equally selected into six groups: normal control group (NC), normal exercise group (NE), high-fat diet group (HFD), high-fat diet plus exercise group (HFE), miR-34a overexpression group (OE), and miR-34a overexpression plus exercise group (OEE). Live morphology showed that treadmill exercise intervention for 8 weeks reduced high-fat diet-induced liver steatosis in mice. 8-week treadmill exercise directly decreased mir-34a expression of mice in HFD group, confirmed in OE group. More, treadmill exercise enhanced the expression of PPARα and SIRT1, thereby affecting the downstream hepatic steatosis-associated target genes, including CPT1(Carnitine palmitoyltransferase 1), CPT2(Carnitine palmitoyltransferase 2), SLC27A1(Solute carrier family 27 member 1), SLC27A4(Solute carrier family 27 member 4), in addition to activating the expression of the central metabolic sensor AMPK. Following aerobic exercise intervention, miR-34a was downregulated, thereby affecting the expression of genes associated with hepatic steatosis, and this mechanism was confirmed in miR-34a overexpression mice. This study contributed to our understanding of the pathogenesis of hepatic steatosis and may provide new therapeutic approaches.

## 1. Introduction

Liver steatosis was the appearance of fat drips in the cytoplasm of liver cells whose long-term development may cause hepatocyte necrosis, liver fibrosis, steatohepatitis which may develop into liver cirrhosis and hepatocellular carcinoma [1,2]. In recent years, with improving of the people’s living standard and decreasing exercise time, liver steatosis has become a serious public health problem in the world, affecting 30–40% of the total population which was still increasing [3]. However, the mechanism of hepatic steatosis was extremely limited and has not been determined. Therefore, the investigation of the molecular mechanisms underlying the progression of hepatic steatosis was urgent in order to obtain effective therapeutic approaches.

MicroRNA (miRNA) was a small single-stranded non-coding RNA of about 19–25 nucleotides in length, which may regulate the expression of its target gene through reverse complementary pairing with the partial sequence of the 3’ untranslated region (3’UTR) of its target gene [4]. In many diseases, miRNAs have received increasing attention due to their imbalance and their potential as both diagnostic and therapeutic targets [5,6]. miR-34a, a specific miRNA, has been reported to show a high level of expression in the hepatic disease patients [7–9]. Besides, studies have reported that inhibition of miR-34a may increase the levels of its downstream targets, including peroxisome proliferator-activated receptor-α (PPARα) and Sirtuin protein family-Sirtuin1 (SIRT1) that were related to fatty acid oxidation thereby alleviating liver steatosis [10].

As a member of the nuclear receptor superfamily, PPARα bound to the ligand form a heterodimer with the retinoid X receptor (RXR), and then the heterodimer bound to the PPAR response element of the target genes promoting the transcription of its downstream genes related to fatty acid oxidation [11]. So PPARα had great effects on the regulation of hepatic lipid metabolism. The key target genes of PPARα downstream that were involved in fatty acid oxidation and fatty acid transport consisted of solute carrier family, including SLC27A and CPTs [12].

SIRT1 was an essential member of the Sirtuin protein family and was a NAD-dependent deacetylase, closely associating with hepatic steatosis [13]. AMP kinase (AMPK), a known regulator of energy metabolism, had a significant role in the development and progression of metabolic diseases, and SIRT1 may activate its activity thus promoting its phosphorylation [14]. Then phosphorylation of AMPK up-regulated β-fatty acid oxidation gene and down-regulated fatty acid transport-related proteins, thereby reducing liver steatosis [15]. Therefore, the SIRT1-AMPK phosphorylation signaling pathway functions in the regulation of lipid metabolic homeostasis and may be a new therapeutic target for hepatic steatosis.

Studies have reported that aerobic exercise was a low-cost, low-risk intervention that may effectively reduce the occurrence and development of liver steatosis and has been adopted by most people [16,17]. The purpose of this study was to determine whether aerobic exercise may improve liver steatosis by adjusting the miR-34a-PPARα/SIRT1 signal pathway.

## 2. Materials and methods

### 2.1 Ethical permission

All operations in this experiment is were performed under the rules of research ethics of Shanxi University with approval number of CIRP-IACUC-(R)2019014, in line with local and international animal ethics guide and with the utmost efforts to minimize animal suffering.

### 2.2 Animal

**Fig 1:**
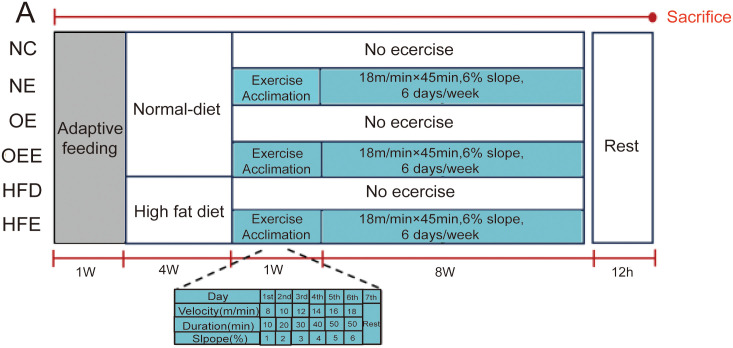
A An overview of the experimental design of the treadmill exercise.

Sixty SPF-grade C57BL/6J male mice (8 week old), weighing 21.4 ± 0.92 g, were bought from Beijing Life River Laboratory Animal Technology Co. (Animal license NO. SCXK (Beijing) 2016–0006). After adaptive feeding for one week, mice were randomly and equally selected into six groups: normal control group (NC), normal exercise group (NE), miR-34a overexpression group (OE), miR-34a overexpression plus exercise group (OEE), high-fat diet group (HFD, 60% fat, 20% carbohydrate, and 20% protein), and high-fat diet plus exercise group (HFE). All mice were housed under standard laboratory conditions with 12/12 dark light cycles, 20–26°C, 40–60% relative humidity, and free access to food and water. Mice body weights were recorded weekly. After the last treadmill exercise, mice were fasted overnight, anesthetized intraperitoneally with a dose of 80 mg/kg pentobarbital sodium, and then executed. Blood samples were collected in EDTA-containing tube, and centrifuged at 4°C and 3500 rpm for 15 min, then the supernatant was transferred to a new tube and stored at −80°C. The livers were rapidly taken out and washed with cold phosphate-buffered saline (PBS), then some of the liver tissues were cut into the fixative and the remaining liver tissues were saved at −80°C. The protocol was shown in (Fig 1A).

After adaptive feeding, TBG promoter-driven overexpression-miR-34a adeno-associated viral vector TBG (AAV9) (100 μL, 1.95 × 1012 vg/ml) (Sangon Biotech, Shanghai, China) was injected into the tail vein to specifically promote miR-34a expression in the mouse liver.

After 4 weeks of a normal or high-fat diet, mice in the NE, OEE, and HFE groups performed 1 week of adaptive treadmill exercise, followed by 8 weeks of treadmill exercise starting at 7 pm every day.

### 2.3 Triglyceride assay

Triglycerides (TG) was measured at 510 nm on a spectrophotometer (UV-6100s, Mapada, Shanghai, China) by using the kit and strictly following the instructions of the kit.

### 2.4 Histologic analysis

Liver tissue was first fixed in 10% neutral buffered formalin and then stained with hematoxylin-eosin (H&E) to view the level of hepatic steatosis by the microscope.

### 2.5 Real-time quantitative PCR

Total RNA was extracted from livers using a spin column animal total RNA purification kit (Sangon Biotech, Shanghai, China) and was reverse-transcribed using M-MuLV first-strand cDNA synthesis kit (Sangon Biotech, Shanghai, China) according to the manufacturer’s protocol. Real-time quantitative PCR (qPCR) was performed on a LightCycler480 system (Roche, Switzerland) using TB Green premix Ex Taq Ⅱ mix (TaKaRa, Dalian, China). Target gene expression was calculated by the 2^-△△CT method. Gene-specific primers were presented in (Table 1), with internal control of GAPDH.

**Table 1:** Primers for mRNA expression analysis in qPCR.

Genes	Forward (F) primer	Reverse (R) primer
miR-34a	5′-AATCAGCAAGTATACTGCCCT-3′	5′-ACT GGT AGT CTG CAA AAC CAA A-3′
PPARα	5′-AGA GCC CCA TCT GTC CTC TC-3′	5′-CTG ATT AAA AAT GTC TCC ACG AAC AG-3′
SIRT1	5′-CCT GAC TTC AGA TCA AGA GAC GGT A-3′	5′-CAC CAG TGA TGA TGC CAT TCT-3′
CTP1	5’-CTC CGC CTG AGC CAT GAA G -−3′	5′-TCC CAA TGC CGT TCT CAA AAT -−3′
CTP2	5′-CAG CAC AGC ATC GTA CCC A -−3′	5′-GAT GCA CGG GAT CGT GTC T -−3′
SLC27A1	5′-CGC TTT CTG CGT ATC GTC TG -−3′	5’-GAT GAA GAC CCG GAT GAA ACG -−3’
SLC27A4	5’-ACT GTT CTC CAA GCT AGT GCT -−3′	5′-GCT GGT GGT CCA GGG GTC TTA CT -−3′
GAPDH	5′-TCA ACG ACC ACT TTG TCA AGC TCA-3′	

### 2.6 Western blot

The liver tissues were lysed in RIPA lysis buffer, and the protein concentration was determined by the BCA method (Beyotime, Shanghai, China). Proteins of 40 μg were separated by sodium dodecyl sulfate-polyacrylamide gel electrophoresis (SDS-PAGE), then cropped and transferred to PVDF membranes, blocked for 1 hour with 5% nonfat milk. After overnight incubation with primary antibody (1:1000 anti-PPARα, anti-SIRT1, anti-AMPK, anti-CPT1, anti-CPT2, anti-SLC27A1, anti-SLC27A4) (Proteintech Group Inc, Wuhan, China), membranes were washed and incubated with HRP-conjugated secondary antibody (1:5000) (Boster Biotech, Wuhan, China). Then the ECL detection kit (Applygen Technologies Inc, Beijing, China) was used for detection, and imaging was performed on a chemiluminescence meter (ChemiDoc XRS + , Bio-Rad, USA).

### 2.7 Statistical analysis

All data are shown as mean±SD (*n* = 10). Statistical analysis was performed using SPSS 25.0 software. One-way ANOVA was used for multiple comparisons. A two-tailed t-test was used for comparison between two groups. *p* < 0.05 was considered to be statistically significant.

## 3. Results

### 3.1 Treadmill exercise alleviated liver steatosis in mice

**Fig 2:**
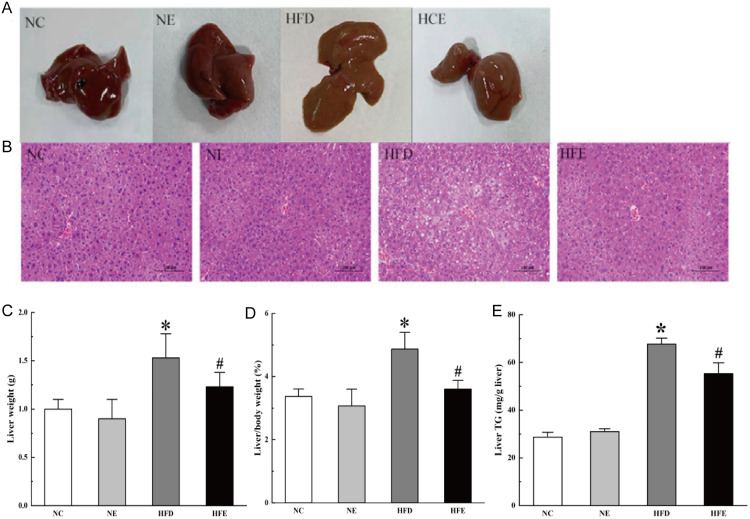
Aerobic exercise prevented HFD-induced hepatic steatosis. (A) Liver morphology. (B) Hepatic lipid droplets. (C) Liver weight. (D) The ratio of liver weight to body weight. (E) Liver TG levels. All data are shown as mean±SD. NC: Normal control group, NE: Normal exercise group, HFD: High-fat diet group, and HFE: High-fat diet plus exercise group. * *p* < 0.05 vs NC, and # *p* < 0.05 vs HFD.

Morphological observation of the liver was showed in (Fig 2A). There were no obvious changes in the liver of NC and NE mice (*p > *0.05), which was fresh red in color with a smooth envelope and clear edges. In The liver of the HFD group mice was yellowish-brown with blunted edges, soft texture, and a greasy surface. After treadmill exercise, the liver status of mice in the HFE group improved compared to mice in the HFD group (*p < *0.05). H&E staining of liver tissue provides an initial assessment of whether steatosis is occurring in the liver. As shown in (Fig 2B), there were no significant changes in the NE group and NC mice (*p > *0.05), while the HFD group showed a significant increase in the number and size of hepatic lipid droplets with inflammatory cell infiltration. In contrast, mice in the HFE group showed a reduced number of hepatic lipid droplets, neatly aligned hepatocytes, and significantly improved hepatic steatosis (*p* < 0.05). Besides, the liver weight (Fig 2C) and the ratio of liver weight to body weight (Fig 2D) of mice in the HFD grou*p* were significantly increased to (1.53 ± 0.25) g (*p* < 0.05) and (4.87 ± 0.53)% (*p* < 0.05) from (1.00 ± 0.10) g and (3.3 ± 0.24)% in the NC grou*p*, respectively, but after aerobic exercise intervention moderately rescued to (1.23 ± 0.15) g (*p* < 0.05 vs HFD grou*p*) and (3.60 ± 0.28)% (*p* < 0.05 vs HFD grou*p*) in the HFE group, correspondingly. One of the major features of liver steatosis was the significant change of liver TG levels, so liver TG levels were detected (Fig 2E). TG levels in the HFD group was significantly increased to 67.67 ± 2.52 (mg/g liver) (*p* < 0.05) com*p*ared with the NC group (28.67 ± 2.08), but after aerobic exercise intervention restored to 55.3 ± 4.53 (mg/g liver) (*p* < 0.05 vs HFD grou*p*) in the HFE group. These observations suggested that high-fat diet-induced hepatic steatosis may be attenuated by treadmill exercise.

### 3.2 Treadmill exercise inhibited miR-34a expression

**Fig 3:**
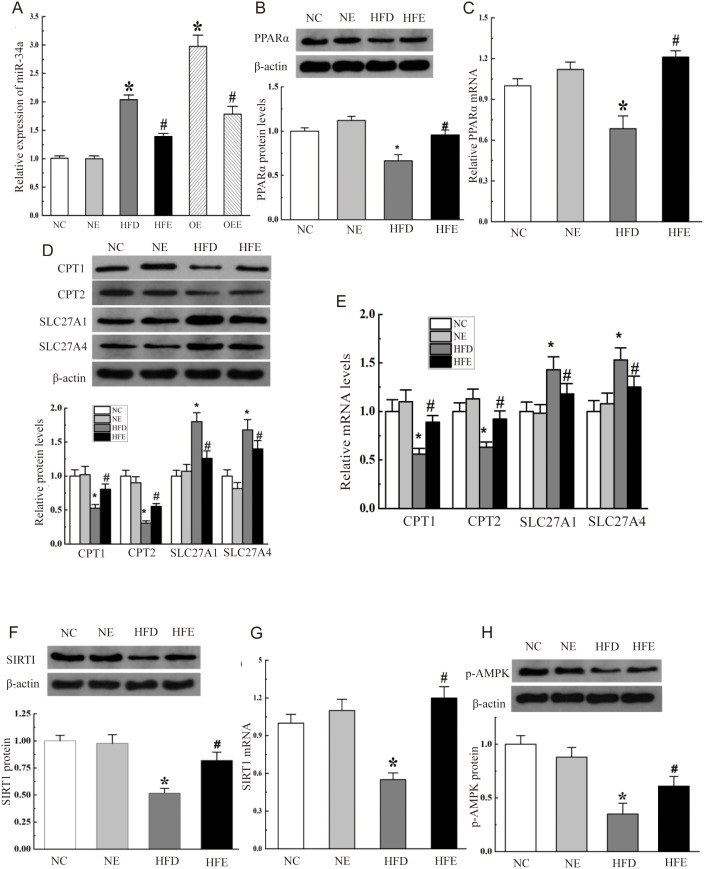
Treadmill training improved hepatic steatosis by inhibiting miR-34a and its downstream targets. (A) Treadmill training inhibited miR-34a mRNA levels increased by high-fat diet and AAV9 injection. (B) The PPARα protein expression was detected in liver tissues by western blotting, with β-actin as a loading control. (C) The mRNA levels of PPARα in mouse liver tissue by qPCR. Internal control: GAPDH. The blots were cropped and the gels were run under the same experimental conditions. (D) Western blotting analysis of PPARα downstream target gene proteins (including CPT1, CPT2, SLC27A4, SLC27A1) in liver tissues. (E) The mRNA levels of CPT1, CPT2, SLC27A4, SLC27A1 in mouse liver tissue by qPCR. (F) The SIRT1 protein expression was detected by western blot. (G) The mRNA levels of SIRT1 in mice liver tissues by qPCR. (H) Western blotting for p-AMPK in liver extracts.OE: miR-34a overexpression group, OEE: miR-34a overexpression plus exercise group.

Next, the effects of treadmill exercise on hepatic miR-34a expression were examined, and the results were showed in (Fig 3A). miR-34a expression levels were not significantly changed in NE group mice compared with NC group mice, whereas miR-34a expression was significantly up-regulated to 2.04 ± 0.08 (*p* < 0.05) compared with the NC group in the liver of HFD mice, and treadmill exercise reversed this rise to 1.39 ± 0.05 (*p* < 0.05). This result revealed that treadmill training could improve liver steatosis by inhibiting miR-34a. To further clarify the effect of aerobic exercise on miR-34a, we injected miR-34a adenovirus vector into mice through the tail vein and performed treadmill intervention. the miR-34a level in the liver of over-expression mice was markedly enhanced to 2.98 ± 0.20 (*p* < 0.05) compared with the NC group, but which was significantly restored to 1.78 ± 0.14 (*p* < 0.05) by aerobic exercise intervention compared with the the Overexpression of miR-34a group, implying that treadmill exercise may down-regulate the expression of miR-34a.

### 3.3 Changes in PPARα and its downstream target genes caused by treadmill exercise

PPARα played an important role in the regulation of hepatic lipid metabolism. The protein expression levels (Fig 3B) and mRNA (Fig 3C) of PPARα in the NE group were basically the same as the NC group, and were decreased to 0.66 ± 0.07 (*p* < 0.05) and 0.69 ± 0.09 (*p* < 0.05) in the HFD group, respectively, while were increased to levels comparable to NC group with 0.96 ± 0.06 (*p* < 0.05) and 1.21 ± 0.05 (*p* < 0.05) after treadmill exercise.

To gain insight into the possible involvement of the miR-34a-PPARα pathway in hepatic steatosis alleviation induced by treadmill exercise, several key target genes of PPARα downstream that involved in fatty acid oxidation and fatty acid transport were examined by qPCR and western blot, including CPT1, CPT2, SLC27A1, and SLC27A4, and the results were shown in (Fig 3D). The expression levels of these downstream target genes in NE group mice were similar to those in NC group mice, while the expression of CPT1 and CPT2 was lower in HFD mice than in NC mice (0.53 ± 0.05 vs. 1.00 ± 0.09, *p* < 0.05 and 0.31 ± 0.04 vs. 1.00 ± 0.07, *p* < 0.05) and the expression of SLC27A1 and SLC27A4 was higher (1.8 ± 0.13 vs. 1.00 ± 0.08, *p* < 0.05 and 1.68 ± 0.15 vs. 1.00 ± 0.09, *p* < 0.05), finally, in the HFE group, the expression of CPT1 and CPT2 was reversed up to 0.81 ± 0.08 and 0.55 ± 0.04 (*p* < 0.05), and the expression of SLC27A1 and SLC27A4 was reversed down to 1.26 ± 0.11 and 1.43 ± 0.12 (*p* < 0.05). Additionally, the mRNA changes of all the above-mentioned genes were confirmed by qPCR (Fig 3E). Thus, the change in PPARα and its target genes could explain the alleviating effect of aerobic exercise on HFD-induced liver steatosis.

### 3.4 SIRT1 was involved in the miR-34a-induced lipid metabolism pathway

As one of miR-34a downstream target gene, SIRT1 was able to modulate the activity of AMP kinase (AMPK), a regulator of energy metabolism. The increase of the AMPK phosphorylation level was closely related to the enhancement of lipid metabolism. Then SIRT1 and AMPK were examined, and (Fig 3F-3G) showed the results. SIRT1 protein levels and mRNA in NE mice were observed no significant changes, and exhibited a significant decrease in mice fed a high-fat diet were found compared to NC mice, with 0.52 ± 0.04 and 0.55 ± 0.05, respectively (*p* < 0.05). Then, this reduction was again up-regulated to 0.82 ± 0.08 and 1.2 ± 0.09, respectively (*p* < 0.05 vs HFD group) after aerobic exercise intervention.

As was shown in (Fig 3H), the level of p-AMPKα (Thr-172) was lower in the HFD group (0.35 ± 0.10, *p* < 0.05) than that in the NC group, while in the HFE group treadmill training partially abolished the decrease of HFD-induced p-AMPK (thr-172) expression (0.61 ± 0.09, *p* < 0.05). Thus, these observations indicated that SIRT1 may involve in the miR-34a-induced lipid metabolism pathway and aerobic exercise may also improve liver steatosis via the miR-34a-SIRT1-AMPK pathway.

### 3.5 Aerobic exercise improved liver steatosis via miR-34a

Changes in miR-34a expression showed a profound regulation of the PPARα/SIRT1-AMPK signaling pathway, which is closely associated with disorders of hepatic lipid metabolism. This relationship prompted us to further investigate whether the PPARα/SIRT1-AMPK pathway was involved in miR-34a-regulated hepatic steatosis and its associated pathology. This was investigated by injecting miR-34a adenovirus vector into mice through the tail vein and then performing 5 weeks of treadmill intervention.

**Fig 4:**
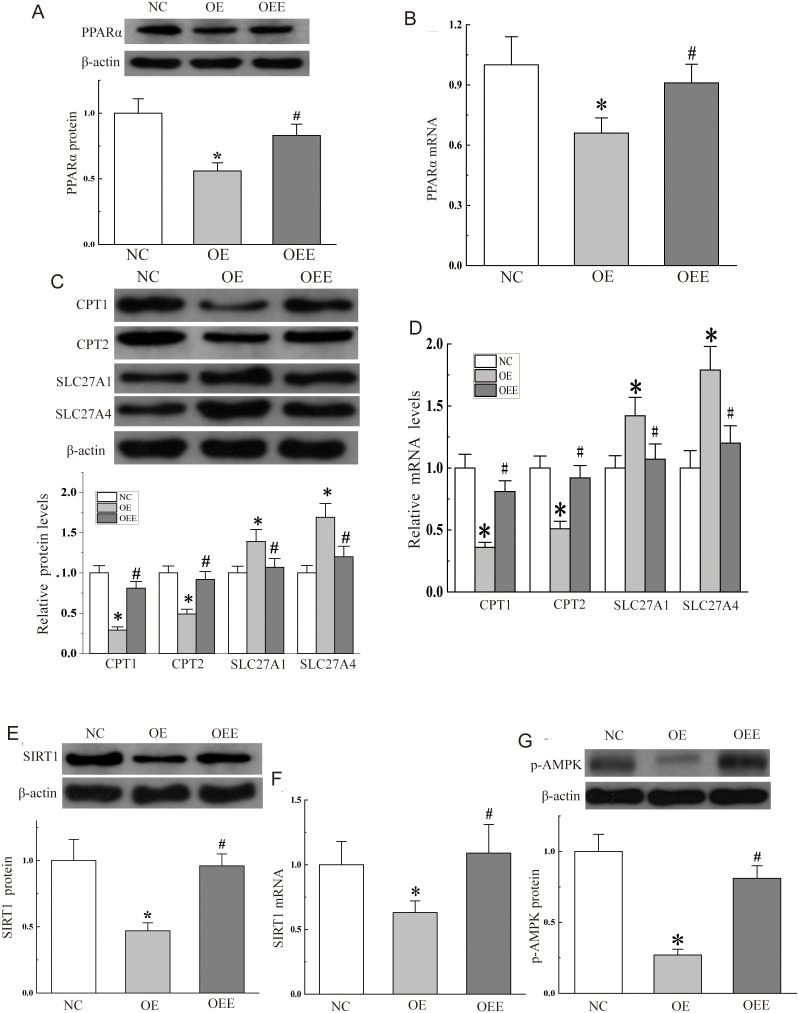
Aerobic exercise improved hepatic steatosis via miR-34a induced by AAV9 injection. (A) Western blot analysis of PPARα protein expression levels and (B) the mRNA level of PPARα. (C) Protein expression levels of downstream fatty acid oxidation and transport-related target genes. (D) mRNA levels of downstream fatty acid oxidation and transport-related target genes. (E) The protein expression levels SIRT1 and (F) the mRNA levels of SIRT1 were assessed by western blot and qPCR, respectively. (G) The phosphorylation levels of AMPK.

As expected, it was observed that treadmill exercise up-regulated the protein expression levels and mRNA of PPARα which both were decreased by miR-34a over-expression (Fig 4A-4B). Also the protein expression levels and mRNA of CPT1 and CPT2 were significantly reduced in liver samples from miR-34a overexpressing mice, and those of SLC27A1 and SLC27A4 were significantly increased, and these changes were altered after the exercise intervention (Fig 4C-4D). It was found in (Fig 4E-4F) that SIRT1 showed the same trend as PPARα. In addition, AMPK phosphorylation levels (Fig 4-4G) were reduced in miR-34a overexpressing mice, and the reduction was significantly reversed after 5 weeks of aerobic exercise. In a word, PPARα/SIRT1-AMPK pathway was involved in miR-34a-regulated hepatic steatosis.

**Fig 5:**
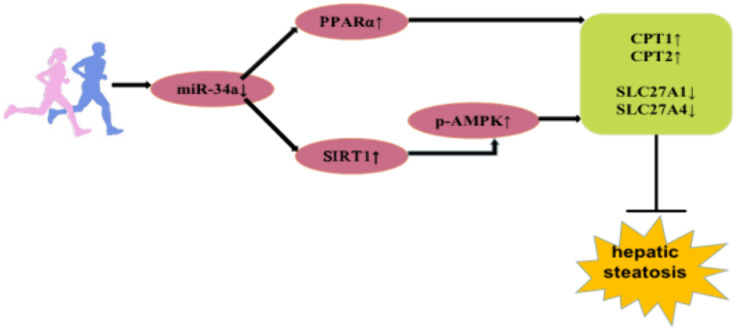
Diagram of the regulatory role of miR-34a-PPARα/SIRT1 on liver steatosis.

The regulatory role of miR-34a-PPARα/SIRT1 in liver steatosis was summarized in (Fig 5).

## 4. Discussion

In the work, after 8 weeks of treadmill exercise, mice in HFD group showed significant improvement in liver weight and liver pathology. Recently, many studies have revealed that miR-34a was a specifically regulated miRNA in liver diseases and has a significant effect on the development and process of hepatic steatosis [10]. The expression level of miR-34a was high in patients with nonalcoholic fatty liver [18,19] as well as in animal models of fatty liver [20,21]. Similarly, our hepatic steatosis mouse model showed increased hepatic expression of miR-34a, which was in agreement with previous findings.

Numerous studies have shown that the expression of miRNA may be affected by aerobic exercise [22]. miR-34a over-expression disrupted lipid normal metabolism and may also be another critical marker for exercise to improve hepatic steatosis. In the work, we showed that treadmill exercise inhibited miR-34a over-expression in HFD mice to reduce hepatic lipid droplet accumulation. Consistent with the morphological findings, aerobic exercise also reduced the level of TG in the liver tissue of mice in the HFE group. Besides, the liver weight/body weight ratio was also markedly reduced in the HFE group when compared to the HFD group. In a word, aerobic exercise inhibited increase of miR-34a, TG, and the liver weight in HFD mice.

miR-34a can potentially post-transcriptionally regulate PPARα through specific binding with the PPARα wild-type luciferase construct [10]. By observing the mRNA and protein levels of PPARα in mouse liver, we found that PPARα mRNA and protein levels were significantly lower in the HFD group compared to the NC group, and 8 weeks of aerobic exercise reversed this decline. As one of the direct downstream target genes of miR-34a, SIRT1 has taken a pivotal role in regulating cell lipid metabolism and reducing inflammation [23–25]. We found that the mRNA and protein levels of SIRT1 were significantly increased in the liver of the HFE group after aerobic exercise. AMPK was an AMP-dependent protein kinase which was widely expressed in various organs related to metabolism and can be activated by exercise, also being a biological energy key molecule of metabolic regulation [11,12]. Our research showed that treadmill exercise increased the expression of SIRT1 through inhibiting miR-34a, thereby stimulating AMPK signals which was to monitor the fuel meter of cell energy status and activate the gene that was related to fatty acid oxidation. Therefore, we supposed that miR-34a-mediated modulation of hepatic lipid metabolism by PPARα and SIRT1-AMPK pathways may be two independent regulatory mechanisms in fatty liver.

CPT1 catalyzed the esterification of long-chain acyl-CoA into L-carnitine which was used as a carrier to transfer long-chain fatty acids from outside the mitochondria to the inside of the mitochondria in the form of an acyl-carnitine conjugate. Then acyl-carnitine conjugates were transformed back to acyl-CoA esters by CPT2 in the mitochondria to promote the oxidation and decomposition of fatty acids [26]. In the work, it was found that both the activation of AMPK and the rise in PPARα expression increased the levels of CPT1 and CPT2, leading to increased transport of fatty acids into the mitochondria, and facilitated the oxidation of mitochondrial palmitoyl-CoA. These observations helped us to understand the mechanism of enhanced fatty acid oxidation after aerobic exercise inhibits miR-34a, with AMPK as a medium for the increased hepatic oxidation of miR-34a after aerobic exercise inhibition.

SLC27 was an integral transmembrane protein that promoted the uptake of long-chain fatty acids into cells and also served as a key player in lipid metabolism [27,28]. Our study showed that the mRNA and protein levels of SLC27A1 and SLC27A4 were significantly elevated in HFD mice, which was significantly reversed after aerobic exercise. This result suggested that aerobic exercise may improve hepatic steatosis by affecting fatty acid transporter proteins.

In conclusion, our research showed that aerobic exercise down-regulated miR-34a, and up-regulated the levels of PPARα and SIRT1 which then affected fatty acid oxidation-related genes CPT1, CPT2, and fatty acid transportation-related genes SLC27A1, SLC27A4 transcription, thereby ameliorated hepatic steatosis. Notably, SIRT1 influenced downstream target genes after activation of the AMPK pathway. This study provided new insights into the pathogenesis of liver steatosis and had the potential to suggest new approaches to treat liver steatosis at the miRNA level.

## Supporting information

S1 Original data. (XLSX)

S2 Raw images. (PDF)
