# Supplementary material for: Aerobic exercise improved liver steatosis by modulating miR-34a-mediated PPARα/SIRT1-AMPK signaling pathway
Source: PLoS One. 2025 Nov 12;20(11):e0333872. doi: 10.1371/journal.pone.0333872 (PMC12611108; doi:10.1371/journal.pone.0333872)

The images of the original blots:

Figure 3.B

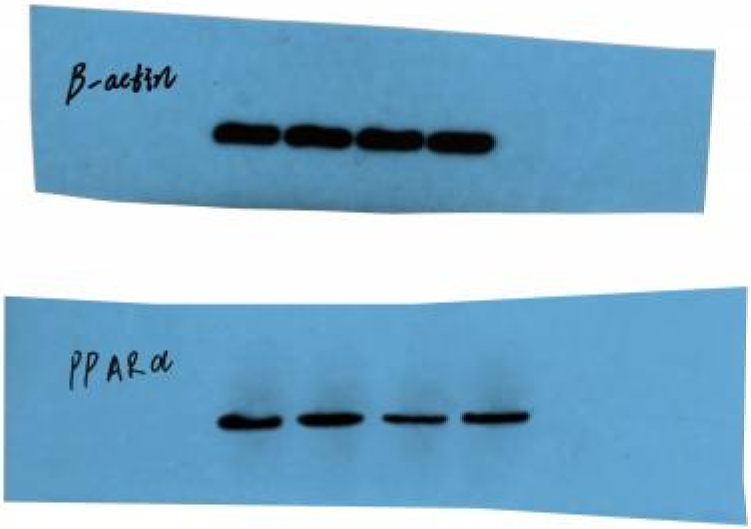

Figure 3.D

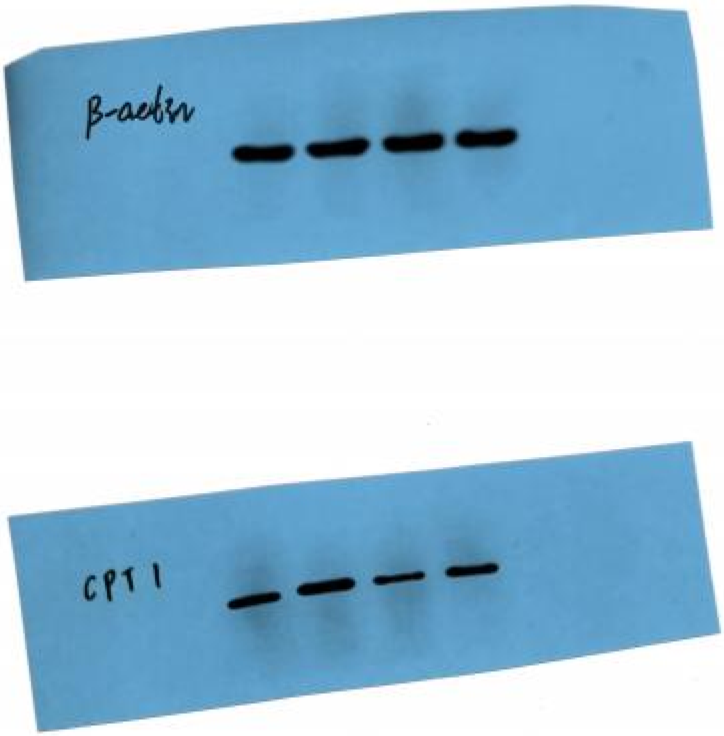

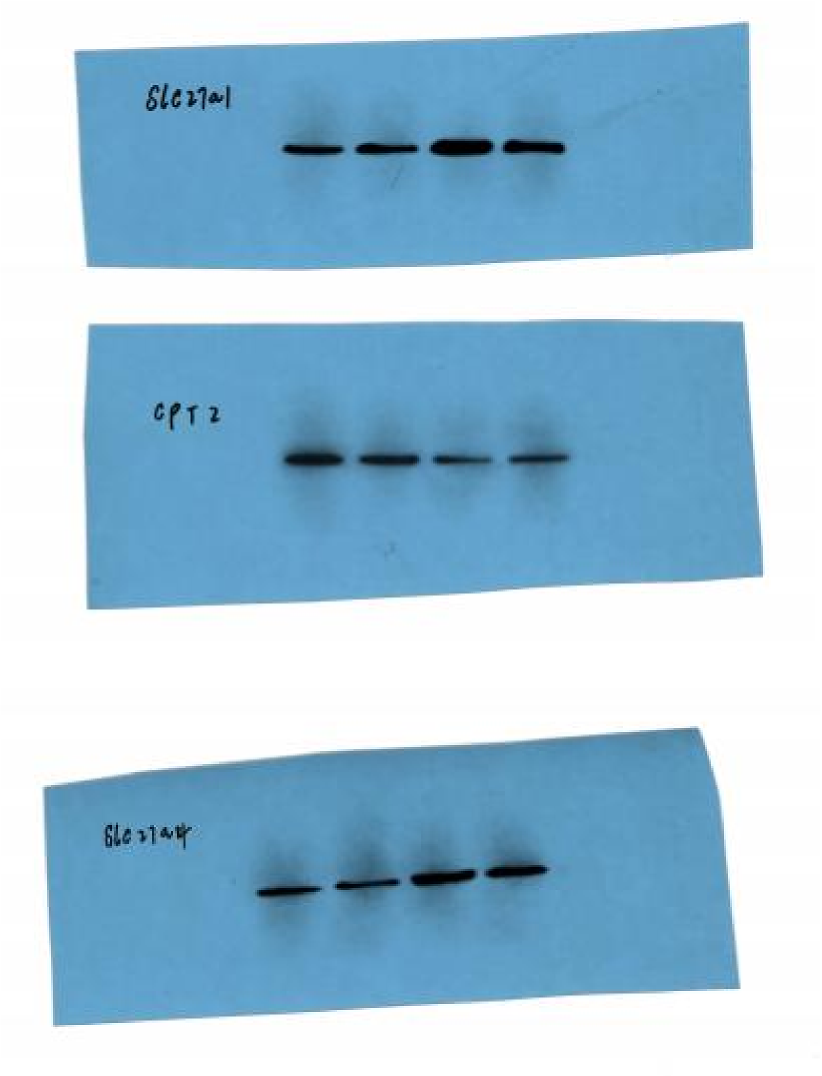

Figure 3.F

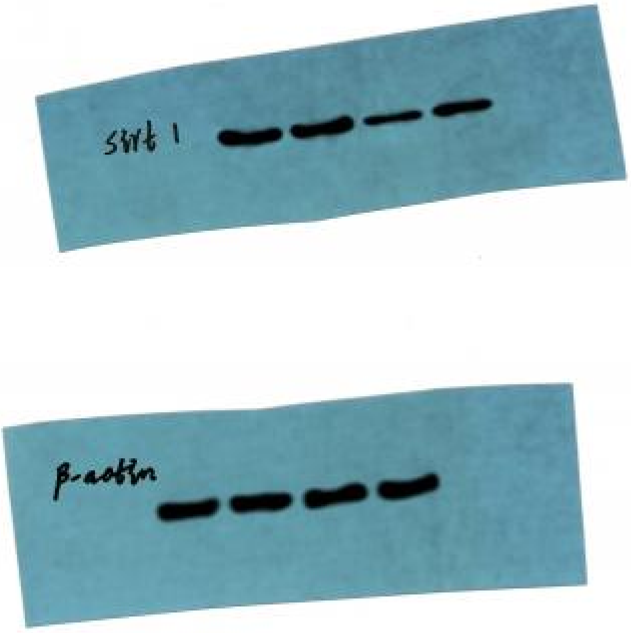

Figure 3.H

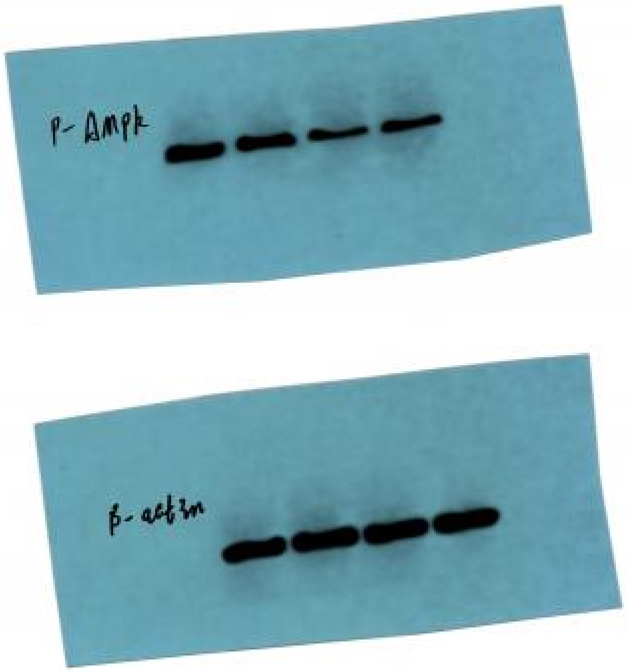

Figure 4.A

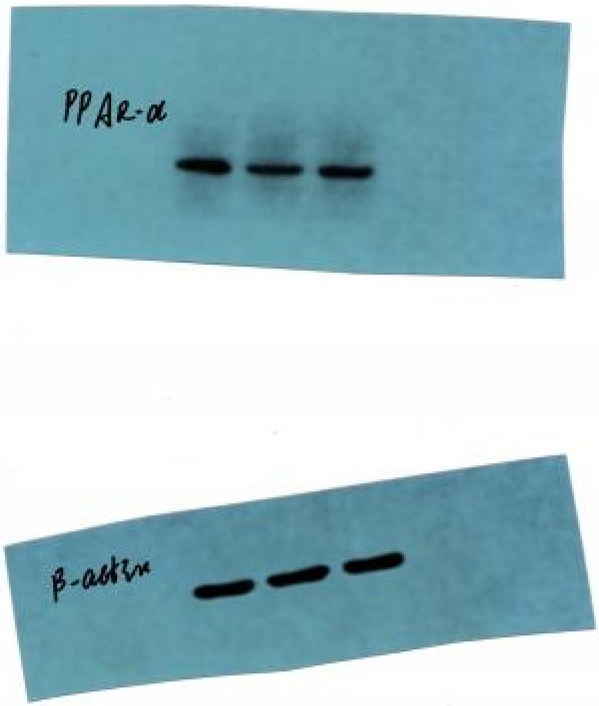

Figure 4.C

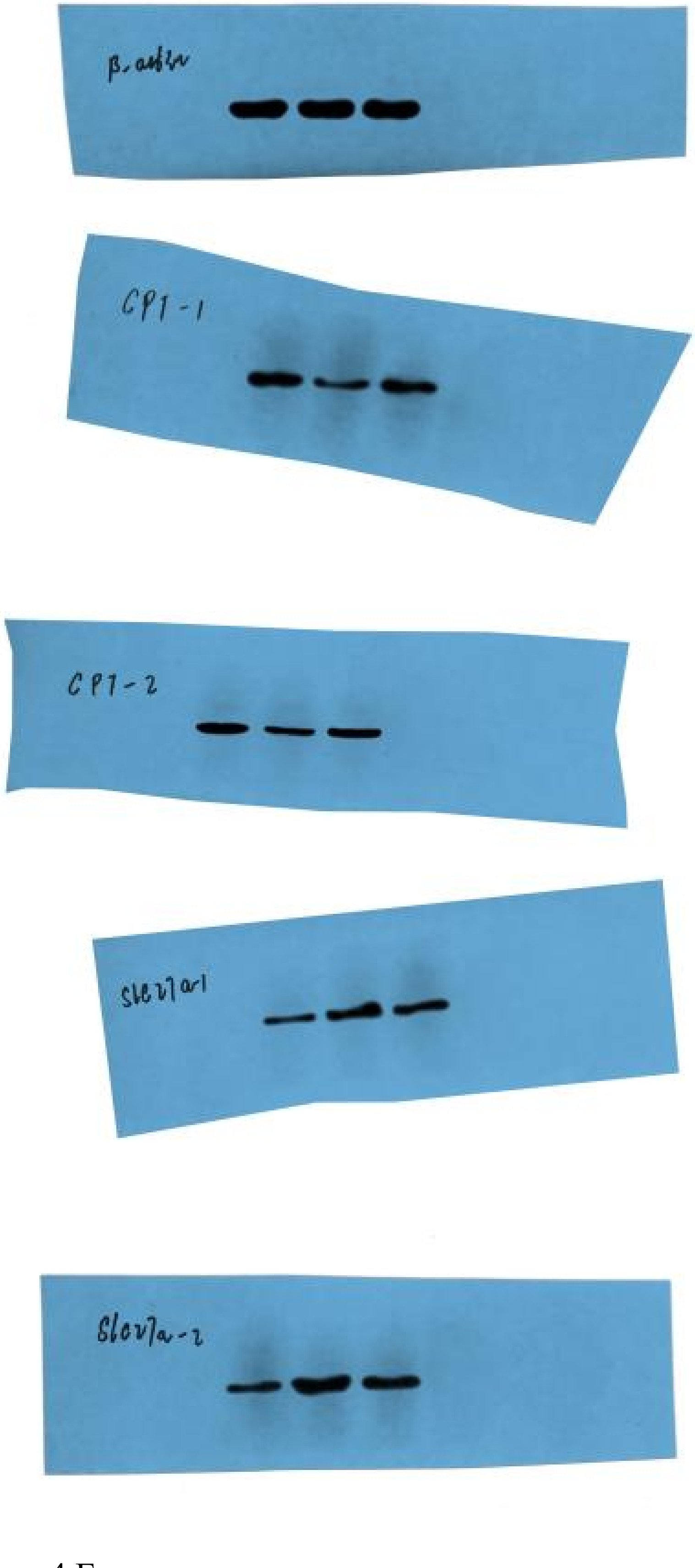

Figure 4.E

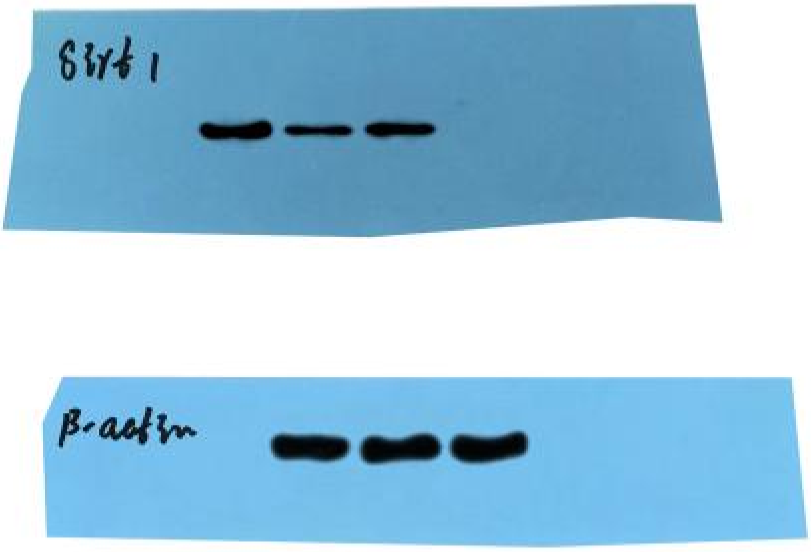

Figure 4.G

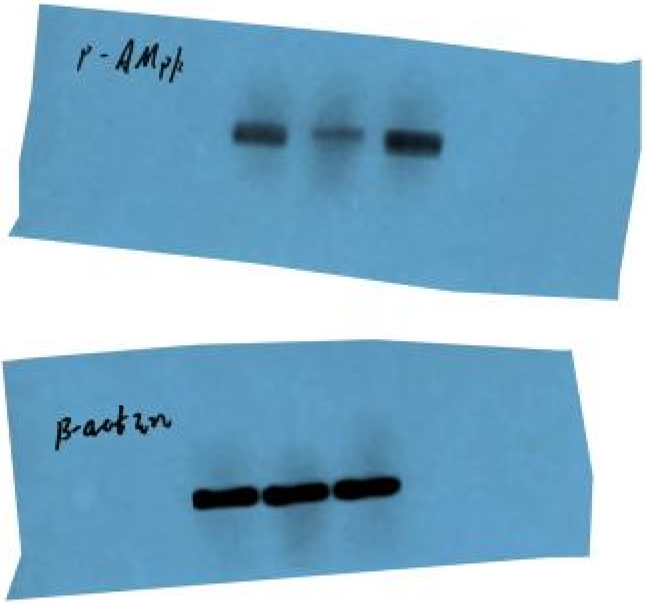

Supplement: S2 — (PDF) [file pone.0333872.s002.pdf]
